# Soluble interleukin-1 receptor type 2 plasma levels in Parkinson’s disease: relationship with cardiac autonomic profile before and after peripheral mechanical somatosensory stimulation

**DOI:** 10.3389/fphys.2023.1168652

**Published:** 2023-08-16

**Authors:** Dana Shiffer, Antonio Roberto Zamunér, Maura Minonzio, Mara Bulgheroni, Alberto Porta, Roberto Leone, Barbara Bottazzi, Cecilia Garlanda, Francesco Colotta, Franca Barbic, Alberto Mantovani, Raffaello Furlan

**Affiliations:** ^1^ Department of Biomedical Sciences, Humanitas University, Milan, Italy; ^2^ Internal Medicine, IRCCS Humanitas Research Hospital, Rozzano, Italy; ^3^ Departamento de Kinesiología, Universidad Católica Del Maule, Talca, Chile; ^4^ Department of Medicine, ASST Fatebenefratelli Sacco, Milan, Italy; ^5^ Department of Biomedical Sciences for Health, University of Milan, Milan, Italy; ^6^ Department of Cardiothoracic, Vascular Anesthesia and Intensive Care, IRCCS Policlinico di San Donato, San Donato Milanese, Italy; ^7^ IRCCS Humanitas Research Hospital, Milan, Italy; ^8^ The William Harvey Research Institute, Queen Mary University of London, London, United Kingdom

**Keywords:** soluble interleukin-1 receptor type 2 (sIL-1R2), cardiovascular autonomic control, Parkinson’s disease, mechanical somatosensory stimulation, heart rate variability

## Abstract

**Introduction:** Systemic inflammation promotes neurodegeneration in Parkinson’s disease (PD). Interleukin-1 receptor type 2 (sIL-1R2) plasma levels increase during inflammation. Data on sIL-1R2 in PD patients and its relationship with PD cardiac autonomic profile are limited, given the possible anti-inflammatory effect of vagal activation. Previously, automated mechanical peripheral somatosensory stimulation (AMPSS) enhanced cardiac vagal modulation. Objectives were to 1) evaluate sIL-1R2 plasma concentrations in PD patients and healthy controls and 2) investigate the correlations between sIL-1R2 and cardiac autonomic indices obtained by spectrum analysis of heart rate variability before and after AMPSS.

**Methods:** sIL-1R2 plasma levels were assessed in 48 PD patients and 50 healthy controls. Electrocardiogram and beat-by-beat arterial pressure were recorded at baseline and after 5 AMPSS sessions in 16 PD patients.

**Results:** PD patients had higher sIL-1R2 levels than controls. In the PD subgroup, an inverse correlation between sIL-1R2 and HFnu was found. There was a negative correlation between changes induced by AMPSS on HFnu and sIL-1R2.

**Discussion:** Higher sIL-1R2 levels in PD patients reflect the inflammatory dysregulation associated with the disease. In PD patients, higher sIL-1R2 was associated with reduced cardiovagal tone. Increased cardiovagal modulation following AMPSS was associated with lower sIL-1R2 levels in Parkinson’s disease patients, suggesting inflammatory state improvement.

## Introduction

Chronic inflammation significantly contributes to the progression of the neurodegenerative process in Parkinson’s disease (PD) (Rocha et al., 2015). Inflammatory changes in affected brain regions of PD patients have a detrimental effect on neurons (Tansey et al., 2022). Increased levels of pro-inflammatory cytokines in the brain and cerebrospinal fluid may promote neuron degeneration (Tansey et al., 2022). In addition, PD patients were also found to have elevated plasma levels of inflammatory markers compared to healthy subjects (Reale et al., 2009; Qin et al., 2016), which often correlated with disease severity (Menza et al., 2010; Lindqvist et al., 2012).

Interleukin-1 (IL-1) is a cytokine with prominent inflammatory functions in the brain, as well as non-inflammatory roles. It is physiologically expressed in the brain, activates neurons (Huang et al., 2011) and regulates several neurophysiological processes, such as sleep, adult neurogenesis, synaptic plasticity and modulation of long-term potentiation (Liu and Quan, 2018). IL-1 also negatively affects neuronal survival (Liu and Quan, 2018) and plays a crucial role in neurodegenerative processes, including PD, Alzheimer’s disease (AD), acute brain injury, central nervous system (CNS) autoimmunity, post-infectious neuropathology, and febrile convulsions (Khazim et al., 2018; Liu and Quan, 2018; Mantovani et al., 2019). IL-1 activity is highly regulated by a receptor antagonist (IL-1Ra) and by negative regulatory receptors, including the decoy IL-1 receptor type 2 (IL-1R2). IL-1R2, cell-associated or in its soluble form, is an endogenous inhibitor of the pro-inflammatory cytokine IL-1 and is involved in the negative regulation of IL-1 activity (Colotta et al., 1994; Mantovani et al., 1996; Molgora et al., 2018; Supino et al., 2022). A soluble form of IL-1R2 (sIL-1R2) is released in inflammatory conditions and it has been used as a marker of inflammation. Indeed, increased levels of sIL-1R2 were detected in cerebrospinal fluid and peripheral blood in various pathological conditions compared to healthy conditions (Supino et al., 2022), including neuro-inflammatory disorders such as AD (Garlind et al., 1999). However, its presence and relevance in PD have not yet been described.

The autonomic nervous system (ANS) plays a crucial role in regulating the complex interactions between the immune and nervous systems (Tracey, 2002). The release of pro- and anti-inflammatory molecules is partly regulated by the ANS (Tracey, 2002; Pavlov and Tracey, 2004), and cytokines affect both arms of the ANS (Kenney and Ganta, 2014). On the other hand, ANS functional status may remarkably influence inflammation. Indeed, through the vagus afferent and efferent nerve fibers, the parasympathetic nervous system may reduce inflammation at the tissue level (Bonaz et al., 2017; Williams et al., 2019; Bellocchi et al., 2022). In contrast, the role of the sympathetic nervous system in inflammation is controversial, as its activation has been shown to either enhance or dampen the activity of immune cells (Furlan et al., 2006; Nance and Sanders, 2007; Pongratz and Straub, 2014).

Signs and symptoms of ANS dysfunction are common in PD patients which include thermoregulation, gastrointestinal, and blood pressure regulation abnormalities such as orthostatic hypotension and supine hypertension (Poewe, 2008; Sharabi et al., 2021). We and others (Barbic et al., 2007; Heimrich et al., 2021; Li et al., 2021) have assessed the magnitude of cardiac dysautonomia in PD by means of power spectrum analysis techniques of heart rate variability (HRV). Results suggested that both sympathetic and vagal cardiac modulatory activities are impaired compared with healthy control subjects (Barbic et al., 2007; Heimrich et al., 2021; Li et al., 2021).

Mechanical foot stimulation has shown positive effects on various Parkinson’s disease (PD) symptoms, such as improved gait and notable changes in cardiac autonomic profile (Barbic et al., 2014; Brognara and Cauli, 2020). Subsequently, repetitive automated mechanical peripheral somatosensory stimulation (AMPSS) applied to the forefeet of PD patients were found to result in a significant increase in the high-frequency component (HF_RR_) and a reduction in the low-frequency component (LF_RR_) and LF/HF ratio of HRV (Zamunér et al., 2019). This meant enhanced parasympathetic and decreased sympathetic modulations to the sinoatrial node (Zamunér et al., 2019), resulting in a decline in systemic arterial pressure in the supine position (Zamunér et al., 2019).

In the current study, we investigated whether peripheral sIL-1R2 plasma levels acted as a marker of PD-associated inflammation by comparing sIL-1R2 plasma levels between PD patients and healthy controls. Furthermore, we assessed whether sIL-1R2 plasma levels correlated with the changes in the spectral indices of cardiac autonomic modulation induced by AMPSS in PD patients.

## Methods

The current study consisted of two parts.

First part. A total of 98 subjects participated in this part of the study. In patients with PD, sIL-1R2 plasma levels were compared to age and gender-matched healthy controls. Table 1 displays the general characteristics of the PD patient and control groups.

**TABLE 1:** Characteristics of PD patients and healthy controls.

Characteristics	PD patients (*n* = 48)	Controls (*n* = 50)
Age (years)	69.5 ± 9.3	62.9 ± 6.9
Male/Female	31/17	32/18
Hoehn & Yahr stage	2–4	-
UDPRS-III score	25.3 ± 16.6	-
Duration of PD (years)	7.5 ± 3.5	-

UPDRS III, unified Parkinson’s disease rating scale; Data expressed as mean ± SD.

Forty-eight PD patients were recruited from the neurology department outpatient clinic of Humanitas Research Hospital, Milano, Italy. PD was diagnosed based on the UK Parkinson’s Disease Brain Bank criteria (UK PDBB) (Hughes et al., 1992). Clinical stage and disease severity were established using the Hoehn and Yahr (H &Y) stage and the Unified Parkinson’s Disease Rating Scale III (UPDRS III- motor function assessment) (Hoehn and Yahr, 1967). Fifty age- and sex-matched healthy controls were also recruited.

Individuals were excluded from the study if they met any of the following conditions: the presence of coexisting neurodegenerative diseases; dementia and/or psychiatric illnesses; clinical history and/or family history of seizures; cardiovascular diseases (atrial fibrillation, arrhythmias, and coronary disorders); chronic inflammatory diseases; chronic use of anti-inflammatory drugs; diabetes mellitus; existing liver, kidney and/or lung disease; regular use of seizure prone drugs and/or psychiatric drugs; a history of alcohol abuse.

Second part. We conducted a prospective interventional investigation using a single-arm pre-post study design. Here we assessed for any associations between sIL-1R2 plasma levels and cardiac autonomic profile before and after repetitive AMPSS in a smaller subgroup of 23 PD patients. The study involved multiple assessments, including a clinical/baseline evaluation, five consecutive AMPSS sessions, and a final clinical/post-AMPSS assessment. The complexity of the second part of the study protocol significantly limited patient participation, resulting in only 23 out of the original 48 PD patients agreeing to take part. Although the sample size was reduced, we considered it to be roughly representative of the larger PD group, with an inclusion rate of approximately 2:1.

This part of the study was held at the Humanitas Research Hospital autonomic outpatient clinic. A week before baseline recordings, each patient underwent a comprehensive clinical evaluation and was introduced to the stimulator device (Gondola™; Gondola Technologies, Epalinges, Switzerland) and the clinical laboratory environment. Arterial pressure was measured using an automated device (Philips M3046A M3) while patients were in a recumbent position before each AMPSS session. Five patients were excluded at enrollment due to peripheral sensory neuropathy, liver, kidney, lung, or heart diseases, diabetes, or any condition related to autonomic dysfunction. Subsequently, two patients were excluded from the final analysis due to short episodes of atrial fibrillation detected during the electrocardiogram (ECG) monitoring. Ultimately, 16 patients participated in the experimental study. PD treatment was unchanged for 30 days before and throughout the study. In this part of the protocol, we will be partially referring to data (Table 2) that were previously published regarding the effects of AMPSS on cardiovascular autonomic control (Zamunér et al., 2019). All patients were evaluated at baseline and 72 h after the last AMPSS stimulation.

**TABLE 2:** General characteristics, baseline sIL-1R2 plasma levels and cardiac autonomic profiles of the PD patient subgroup (*n* = 16).

Characteristics	PD patients
Age (years)	66 ± 9
Male/Female	6/10
Body mass index (kg/m^2^)	24.2 ± 2.8
Hoehn-Yahr stage	2–4
UDPRS-III score	25.3 ± 16.6
Disease duration (years)	7.0 ± 3.5
Levodopa (mg/day)	249.5 ± 351.6
sIL-1R2 (pg/ml)	8,756.76 ± 2,117.67
SAP (mmHg)	131 ± 5
DAP (mmHg)	77 ± 3
R-R Interval (ms)	898 ± 184
σ^2^_RR_ (ms^2^)	563 ± 507
HF_RR_ (ms^2^)	123.5 ± 136.8
HF_RR_ (nu)	50.2 ± 13.7
LF_RR_ (ms^2^)	138.4 ± 113.7
LF_RR_ (nu)	49.8 ± 13.7
LF/HF	1.56 ± 0.96

UPDRS III, unified Parkinson’s disease rating scale; sIL-1R2, soluble interleukin-1 receptor type 2; SAP, systolic arterial pressure; DAP, diastolic arterial pressure; σ^2^_RR_, variance of R-R interval; HF, high frequency component; LF, low frequency component; LF/HF ratio between the low frequency and the high frequency components of RR variability; nu, normalized units. Data expressed as mean ± SD. Some of the data displayed in the present table had been previously reported (Zamunér et al., 2019).

### Experimental procedure

Automated mechanical peripheral somatosensory stimulation (AMPSS) was applied to the supine patient’s forefeet over two specific points using a device (Gondola™; Gondola Technologies, Lugano, Switzerland). A full description of the device and the required adjustments are provided elsewhere (Zamunér et al., 2019). This shoe-shaped device contains built-in electrical motors activating two rounded-tip steel rods that deliver mechanical pressure over the tip of the hallux and the plantar surface of the first metatarsal joint of the foot. Those specific sites were used based on previous research showing significant changes in the cardiovascular autonomic modulation in PD patients, namely an increased cardiovagal modulation and overall reduced sympathetic activity (Barbic et al., 2014). At enrollment, the magnitude of the applied mechanical pressure was individualized for each patient. It was set as the amount of pressure that elicited the reflex withdrawal (i.e., tibialis muscle contraction) and was subsequently used during all stimulation procedures.

Each participant underwent an AMPSS session every 72 h for a total of 5 sessions. It is important to highlight that while AMPSS proved to reduce blood pressure in PD patients while supine, no orthostatic hypotension nor symptoms of orthostatic intolerance were reported by the patients, in keeping with a previousobservation in a PD population after the same somatosensory stimulation (Zamunér et al., 2019).

For each patient, ECG, noninvasive beat-by-beat blood pressure (Nexfin monitor, BMEYE B.V., Amsterdam, Netherlands), and respiratory activity (thoracic bellow) were continuously recorded for fifteen minutes in the supine position. The signals were digitalized at 300 Hz/signal by an analog-to-digital converter (ADInstruments, Powerlab DAQ, PL3516/P, Oxford, UK) and stored onto a hard disk for offline analysis.

### Cardiac autonomic profile evaluation

The cardiac autonomic profile of the patients was assessed twice during the study: before intervention (baseline) and 72 h after the last AMPSS session. Complete data on the effects of AMPSS on cardiovascular autonomic control are described elsewhere (Zamunér et al., 2019). Notably, the present study is a further extension of a previously published work (Zamunér et al., 2019). Since we are now focused on the relationship between inflammatory markers and cardiac autonomic control and their changes induced by repetitive AMPSS, in the present study we needed to provide the reader with the baseline values of the indices of cardiac autonomic profile, although already published (Zamunér et al., 2019). HRV analysis results are expressed as delta values in Figure 3. Delta values were computed as the difference between HF nu and LF nu values after repetitive AMPSS–HF nu, LF nu and LF/HF Pre AMPSS values, respectively.

Spontaneous fluctuations in heart rate (or in the ECG R waves interval, i.e., RR interval) result from instantaneous influences of sympathetic and parasympathetic modulation on the sinus node activity. Spectral analysis techniques were used to assess the amplitude and frequency of these oscillations. A detailed description of the software techniques for data acquisition, spectral, and cross-spectral analyses of RR interval variability is reported elsewhere (Heart rate variability, 1996; Furlan et al., 2000). Briefly, the high frequency (HF) and low frequency (LF) are the two major oscillatory components obtained from RR interval variability. The former is characterized by a central frequency ranging between 0.15 Hz to 0.4 Hz, and its power, HF_RR_, reflects the vagal (i.e., parasympathetic) efferent modulatory activity directed to the sinoatrial node (Pomeranz et al., 1985; Furlan et al., 2000). The LF_RR_ component is characterized by a central frequency ranging from 0.04 Hz to 0.15 Hz. Its power, when expressed in normalized units (nu), may be used as an index of the sympathetic modulatory activity to the sinoatrial node (Pagani et al., 1986; Furlan et al., 2000), although its physiological role is still debated (Pomeranz et al., 1985; Reyes del Paso et al., 2013). The LF/HF ratio is a dimensionless index that reflects the instantaneous sympathovagal balance (Pagani et al., 1986; Furlan et al., 2000).

### Samples collection and sIL-1R2 measurement

sIL-1R2 plasma levels were checked before the intervention (baseline) and 72 h after the last AMPSS session, i.e., after the 5th stimulation session.

Venous blood samples from the antecubital fossa area were collected in Ethylenediaminetetraacetic Acid (EDTA) containing vacutainer tubes from each subject under fasting conditions. Plasma was obtained by centrifuging blood samples within 45 min of blood withdrawal. The plasma samples were then frozen at −80°C. Plasma concentrations of sIL-1R2 were measured by solid-phase, enzyme-linked immunosorbent assay (ELISA) developed in-house and based on the sandwich principle, as previously described (Müller et al., 2002). Briefly, the monoclonal antibody 8.5 was used as capturing antibody while detection of bound sIL-1R2 was performed using a biotin-labeled polyclonal rabbit serum. Plasmatic levels of sIL-1R2 were calculated by linear regression using a standard curve made with the recombinant purified protein (R&D Systems) as a reference. Changes induced by repetitive AMPSS on sIL-1R2 plasma levels were expressed as delta values. Delta values were computed as the difference between sIL-1R2 plasma levels after Repetitive AMPSS–sIL-1R2 plasma levels Pre AMPSS.

## Statistical analysis

Kolmogorv-Smirnov and Shapiro-Wilk test were applied to assess whether data were normally distributed. A comparison of sIL-1RI2 plasma levels between the PD and control groups was performed using the parametric t-Student test for unpaired data. For the experimental part of the study involving a subgroup of PD patients, the effects of repetitive AMPSS on the cardiac autonomic profile and sIL-1R2 were analyzed by the non-parametric Wilcoxon signed-rank paired test. The Spearman’s correlation test was used to check for relationships between sIL-1R2 plasma levels and cardiac autonomic indices at baseline and following 5 AMPSS sessions. GraphPad Prism™ software was used for statistical analysis. Categorical measurements are reported as number (*n*) and percentage (%). Continuous variables are displayed as mean ± standard deviation (SD). The significance level was set at 5%.

## Study approval

The study protocol was approved by Humanitas Research Institute Ethics Committee (Authorization n.1395) and was registered on clinicalTrials.gov (#NCT02608424). All participants provided written informed consent to participate in the study.

## Results

### sIL-1R2 plasma levels in patients with Parkinson’s disease compared to age and sex-matched healthy controls

Table 1. Displays the general characteristics of the two groups. There were no significant differences between the groups concerning age and sex.

**FIGURE 1:**
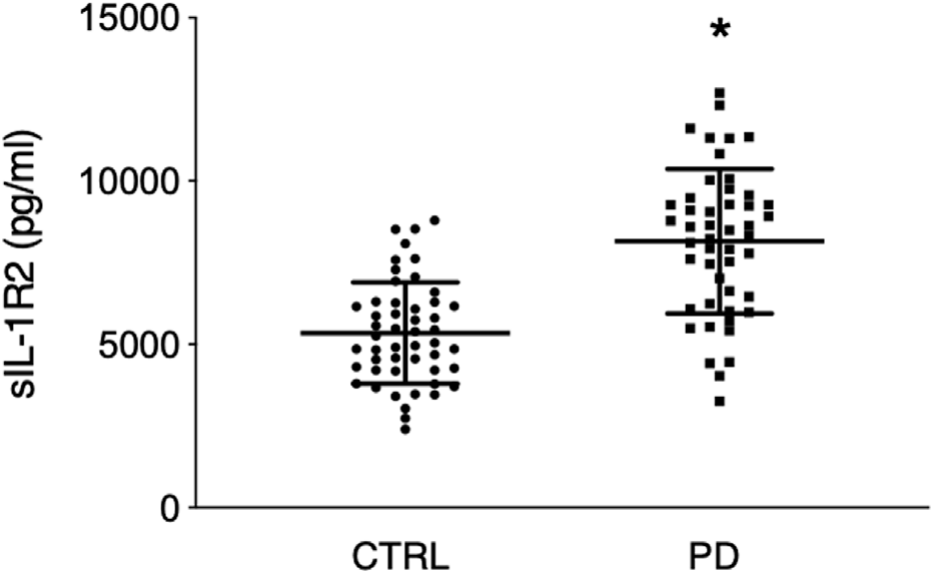
sIL-1R2 plasma levels in control group (*n* = 50) and PD group (*n* = 48). Please note that PD patients were characterized by greater sIL-1R2 plasma values than the control group suggesting the possible presence of an underlying systemic inflammatory state. CTRL indicates control group; PD, Parkinson’s disease group; sIL-1R2, soluble interleukin-1 receptor type 2. The horizontal bars in each column indicate group mean ± SD. *p < 0.0001.

Mean plasma concentration of sIL-1R2 was significantly higher in the PD patient group compared to the healthy control group (8,089 ± 2,213 pg/ml vs. 5,344 ± 1,551 pg/ml, respectively; *p* < 0.0001) (Figure 1).

### Relationships between baseline sIL-1R2 plasma levels and cardiac autonomic profile in the PD patient subgroup

**FIGURE 2:**
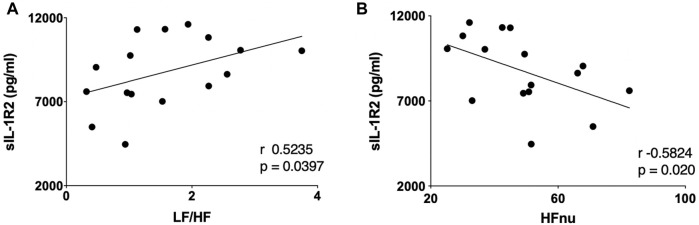
Correlations between sIL-1R2 plasma levels and cardiac autonomic indices, LF/HF and HFnu, at baseline, in the PD subgroup (*n* = 16). Note that the greater the cardiac sympathetic modulation, as suggested by the LF/HF ratio pattern, the greater the sIL-1R2, an index reflecting the magnitude of systemic inflammation **(A)**. Conversely, the greater the cardio-parasympathetic modulatory activity indicated by the HF_RR_ marker, the lower the sIL-1R2 plasma levels **(B)**. sIL-1R2 indicates soluble interleukin-1 receptor type 2; LF/HF, LF/HF ratio of RR variability; HFnu, high frequency component of RR variability expressed in normalized units (nu).

Table 2. Describes the general characteristics, baseline sIL-1R2 plasma level, and cardiac autonomic profiles in the PD patient subgroup. In this subgroup, a significant positive correlation was found between baseline levels of sIL-1R2 and baseline LF/HF ratio (r 0.524, *p* = 0 .040) (Figure 2A). Additionally, before AMPSS, a significant inverse correlation was seen between sIL-1R2 levels and HFnu (r −0.582, *p* = 0 .020) (Figure 2B). These results indicate that higher cardiac sympathetic modulation and lower cardiac parasympathetic modulation were associated with higher plasma levels of sIL-1R2.

### sIL-1R2 plasma level changes and its relationships with cardiac autonomic profile modifications in PD patient subgroup following repetitive AMPSS

**FIGURE 3:**
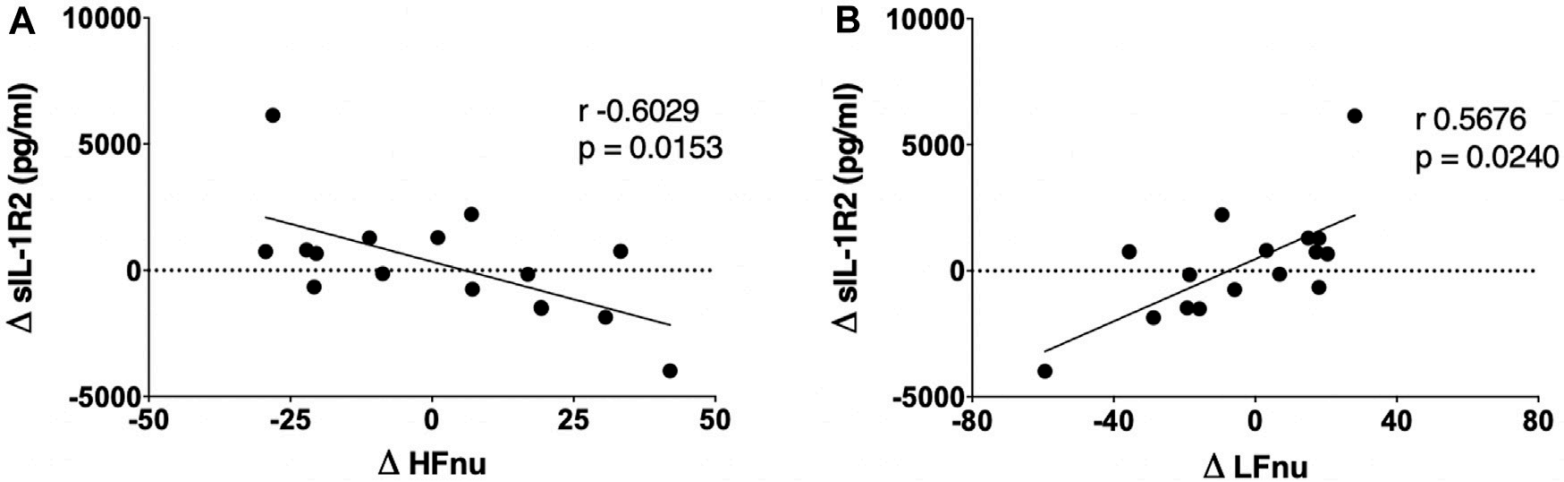
Correlations between modifications in cardiac autonomic indices and changes in sIL-1R2 induced by repetitive AMPSS in a PD subgroup (*n* = 16). The greater the increase of HFnu, a marker of the cardiac parasympathetic modulatory activity, the greater the decrease of sIL-1R2 plasma levels after repetitive AMPSS **(A)**. Additionally, the greater the decrease in LFnu, a marker of the cardiac sympathetic modulation, the greater the reduction of sIL-1R2 plasma levels after repetitive AMPSS **(B)**. sIL-1R2 indicates soluble interleukin-1 receptor type 2; HFnu, high frequency component of heart rate variability in normalized units (nu). LFnu, low frequency component of heart rate variability in normalized units (nu).

Repetitive AMPSS was not associated with a significant reduction in the plasma levels of sIL-1R2 (∆ 188.2 ± 2,126.5 pg/ml) when compared to baseline (8,780.8 ± 2,123.5 pg/ml; *p* > 0.05). However, a significant negative correlation (r = −0.60, *p* = 0.015) between delta sIL-1R2 (∆ 188.2 ± 2,126.5 pg/ml) and delta HFnu (∆ 2.2 ± 23.2) (Figure 3A), and a significant positive correlation (r = 0.57, *p* = 0.02) between delta sIL-1R2 and delta LFnu (∆ −4.1 ± 24.3) (Figure 3B) were seen. These findings suggest that the patients who most reduced sIL-1R2 levels were the ones who most reduced the cardiac sympathetic modulation and increased the cardiac parasympathetic modulation after AMPSS.

## Discussion

The main findings of the present study are that 1) patients with PD had higher sIL-1R2 plasma concentration than age- and sex-matched healthy subjects; 2) in PD patients, higher sympathetic and lower parasympathetic modulatory activities to the heart, as assessed by the spectral markers of RR variability, were associated with higher plasma levels of sIL-1R2, and 3) changes in the spectral indices of cardiac autonomic control, elicited by repetitive AMPSS, were related to changes in the levels of sIL-1R2. Namely the greater the increase in HF_RR_ the greater the reduction in sIL-1R2 plasma levels and similarly, the greater the decline in LF_RR_ the larger the decrease in sIL-1R2 after AMPSS.

The soluble form of the regulatory IL-1 decoy receptor, sIL-1R2, which was markedly higher in our PD patients, was shown to be upregulated in several inflammatory chronic diseases such as multiple sclerosis, rheumatoid arthritis, and AD, acting as an inflammatory biomarker (Molgora et al., 2018; Supino et al., 2022). IL-1R2 mediates anti-inflammatory functions in the periphery and central nervous system by inhibiting the effects of IL-1 (Molgora et al., 2018; Supino et al., 2022), a potent pro-inflammatory cytokine that was found to be elevated in PD patients (Qin et al., 2016) and other neurodegenerative disorders (Mantovani et al., 2019). During inflammatory conditions, sIL-1R2 is released into the bloodstream where it binds to IL-1α and IL-1β, making it a potential biomarker for inflammatory diseases (Supino et al., 2022). However, its role as a potential biomarker or prognostic tool, is still unclear as research findings have been inconsistent. Studies have shown that increased levels of sIL-1R2 are associated with various inflammatory conditions, such as necrotizing enterocolitis, acute respiratory distress syndrome, acute meningococcal infection, and sepsis (Supino et al., 2022). In many of these cases, sIL-1R2 levels were found to reflect the severity of the disease. However, in other contexts, such as rheumatoid arthritis, the concentration of sIL-1R2 negatively correlated with the severity of the disease, indicating that endogenous sIL-1R2 may function as a natural anti-inflammatory factor in chronic polyarthritis (Jouvenne et al., 1998). In multiple sclerosis patients, sIL-1R2 levels increased in cerebrospinal fluid following steroid therapy, suggesting a potential beneficial effect of the molecule (Dujmovic et al., 2009). Finally, a previous study that showed increased sIL-1R2 levels in the cerebrospinal fluid of AD patients proposed that it might reflect a compensatory anti-inflammatory mechanism attempting to inhibit IL-1 receptor-mediated activity in the brain (Garlind et al., 1999). PD, like AD, is characterized by a chronic neurodegenerative process with a substantial inflammatory component. Thus, it is plausible that the higher sIL-1R2 plasma levels in our PD patients compared to the healthy controls might reflect PD associated inflammation and the presence of a similar compensatory mechanism aimed to counterbalance the IL-1 pro-inflammatory effects in the disease (Mantovani et al., 2019).

The current study results showed that, before AMPSS, higher levels of sIL-1R2 were associated with lower HF_RR_ nu values, an index of cardio-vagal (i.e., parasympathetic) modulation, and higher LF/HF ratios, indicating a shift in the sympathovagal balance towards a relative sympathetic predominance. This implies that lower parasympathetic modulation was associated with a higher degree of inflammation in our group of PD patients. In keeping with this finding are the results of several studies including a recent meta-analysis performed on 51 clinical studies assessing the relationship between HRV indices derived from frequency and time domain analyses and markers of inflammation (Aeschbacher et al., 2017; Williams et al., 2019; Badke et al., 2022). A general negative association was found between indices of parasympathetic-mediated HRV and markers of inflammation in accordance with our observations. Moreover, for the frequency domain, the HF_RR_ nu index showed more robust and consistent negative associations across markers of inflammation (Williams et al., 2019). In addition, it was found that COVID-19 patients experienced significant drops in HRV, indicating decreased parasympathetic activity, prior to an increase in the acute inflammatory marker C-reactive protein (Hasty et al., 2020). This observation suggests that HRV could potentially serve as a predictive marker for acute inflammatory response in individuals diagnosed with COVID-19 (Hasty et al., 2020).

Chronic inflammatory conditions were found to be associated with increased sympathetic nerve activity (Furlan et al., 2006; Nance and Sanders, 2007; Pongratz and Straub, 2014). Additionally, studies have demonstrated the association between increased sympathetic tone and inflammatory cytokine levels (Pongratz and Straub, 2014). This is also noted in our PD patients, since those with increased cardiac sympathetic modulation, as assessed by enhanced LF/HF ratio, also had higher plasma levels of sIL-1R2. As previously mentioned, higher circulating sIL-1R2 levels are present in inflammatory processes (Supino et al., 2022), and IL-1β is increased in PD patients (Qin et al., 2016). Microglial cells in the brain upregulate IL-1R2 in different pathological conditions of the central nervous system and in response to IL-1β injected into rat brains (Docagne et al., 2005; Supino et al., 2022). In other animal studies, IL-1β administration was shown to increase sympathetic drive through the activation of the central nervous system (Ericsson et al., 1997) and resulted in increased blood pressure and heart rate (Yao et al., 2020).

Regarding the effects of AMPSS on sIL-1R2 plasma levels, our hypothesis is based on the parasympathetic regulation of inflammation through the cholinergic anti-inflammatory pathway (Pavlov et al., 2003; Pavlov and Tracey, 2005; Tracey, 2007), since previous studies have shown that AMPSS is effective in shifting the cardiovascular autonomic control towards a greater cardiac parasympathetic and reduced sympathetic modulatory activities in patients with PD (Barbic et al., 2014; Zamunér et al., 2019). Although five AMPSS sessions could not significantly modify sIL-1R2 plasma levels in our PD patients, we found significant correlations between changes in cardiac autonomic control spectral indices and changes in this inflammatory marker. Specifically, patients who most decreased their sIL-1R2 levels were those who most increased the cardiac parasympathetic and decreased cardiac sympathetic modulation after AMPSS.

The autonomic nervous system plays a major role in the regulation of inflammation. The cholinergic anti-inflammatory pathway modulates inflammation through a parasympathetic reflex that involves a brainstem integrated communication between vagal afferent and efferent nerve fibers (Pavlov and Tracey, 2005). Following an acute inflammatory response afferent fibers of the vagus nerve may transmit information to the solitary nucleus tract. Subsequently, activation of vagal efferent fibers inhibits the production of pro-inflammatory cytokines through the neurotransmitter acetylcholine (Tracey, 2002; Andersson and Tracey, 2012). Some authors have challenged this model by showing that inflammation is modulated by an inhibitory neural reflex mediated by sympathetic nerves rather than by vagus nerves (Singh et al., 2009; Martelli et al., 2014). In one of those studies, lipopolysaccharide treatment in rats increased efferent activity in the greater splanchnic nerve through conventional sympathetic nerve pathways, and suppressed plasma TNFα response by 80% (Martelli et al., 2014). Moreover, vagotomy had no discernible effect on reflex control of inflammation (Martelli et al., 2014). However, recently the cholinergic anti-inflammatory pathway hypothesis was supported by a large meta-analysis that found a general negative association between HRV, namely HF-HRV, and markers of inflammation (Williams et al., 2019).

## Limitations

We acknowledge the following limitations of the current study.

First, we did not perform a randomized controlled trial (RCT) to check the effects of repetitive AMPSS on the inflammatory marker sIL-1R2. Rather the second part of the current study was designed as a prospective interventional investigation, with a single arm pre-post intervention. Indeed, due to the strenuous nature for PD patients of the AMPSS protocol and because of the previously observed beneficial effects of AMPSS both on gait and blood pressure in PD patients (Brognara and Cauli, 2020; Heimrich et al., 2021), we speculated that a randomization procedure was to some extent unethical, would have remarkably limited the sample size of each of the two resulting groups (i.e., the placebo and treated individuals) and would have eventually had a negative impact on the study feasibility.

Second, the main aim of the second part of the curreny study was to assess the association between the cardiac autonomic profile changes elicited by AMPSS, and possible concomitant modifications in the IL-1R2 plasma levels resulting by the activation of the cholinergic anti-inflammatory pathway. However, because of the current study design, we could not infer any causality. While we had the strength of temporality, suggesting that outcomes may be related with the intervention, on the other hand the observed modifications in both sIL-1R2 plasma levels and cardiac autonomic profile indices could not be fully attributed to the AMPSS.

Finally, the mechanistic relationship between neuroinflammation, sIL-1R2 and cardiovagal modulation were only partially assessed in the current study since the linear relationships that were found did not give us any causality information. Future *ad hoc* studies should examine the association between inflammatory biomarkers, symptom severity, disease progression, and their role in prognosis and potential therapeutic strategies in patients with PD.

Because of the above limitations, the present study has to be regarded as a “proof of concept” for future RCTs to check the effects of AMPSS on inflammation.

## Conclusion

The current study first part results showed that PD patients had higher sIL-1R2 plasma levels than healthy controls, providing additional evidence of the possible presence of peripheral inflammatory dysregulation in the disease. Moreover, before AMPSS was applied, a significant inverse correlation was found between the level of sIL-1R2 and cardiac parasympathetic tone in PD patients. Although 5 AMPSS sessions did not significantly change circulating sIL-1R2 levels, there was a significant association between sIL-1R2 plasma levels and autonomic nervous system spectral indices. Specifically, patients who most increased the cardiac parasympathetic and decreased cardiac sympathetic modulation indices after AMPSS were those who most decreased the marler of systemic inflammation sIL-1R2 after AMPSS, possibly suggesting an improvement in their overall inflammatory state.

## Data Availability

The raw data supporting the conclusion of this article will be made available by the authors, without undue reservation.

## References

[B1] Aeschbacher S. Schoen T. Dörig L. Kreuzmann R. Neuhauser C. Schmidt-Trucksäss A. (2017). Heart rate, heart rate variability and inflammatory biomarkers among young and healthy adults. Ann. Med. 49 (1), 32–41. 10.1080/07853890.2016.1226512 27534940

[B2] Andersson U. Tracey K. J. (2012). Reflex principles of immunological homeostasis. Annu. Rev. Immunol. 30, 313–335. 10.1146/annurev-immunol-020711-075015 22224768 PMC4533843

[B3] Badke C. M. Carroll M. S. Weese-Mayer D. E. Sanchez-Pinto L. N. (2022). Association between heart rate variability and inflammatory biomarkers in critically ill children. Pediatr. Crit. Care Med. 23 (6), e289–e94. 10.1097/pcc.0000000000002936 35293369

[B4] Barbic F. Galli M. Dalla Vecchia L. Canesi M. Cimolin V. Porta A. (2014). Effects of mechanical stimulation of the feet on gait and cardiovascular autonomic control in Parkinson's disease. J. Appl. Physiol. 116(5), 495–503. 10.1152/japplphysiol.01160.2013 24436294

[B5] Barbic F. Perego F. Canesi M. Gianni M. Biagiotti S. Costantino G. (2007). Early abnormalities of vascular and cardiac autonomic control in Parkinson's disease without orthostatic hypotension. Hypertension 49 (1), 120–126. 10.1161/01.hyp.0000250939.71343.7c 17101845

[B6] Bellocchi C. Carandina A. Montinaro B. Targetti E. Furlan L. Rodrigues G. D. (2022). The interplay between autonomic nervous system and inflammation across systemic autoimmune diseases. Int. J. Mol. Sci. 23 (5). 10.3390/ijms23052449 PMC8910153 35269591

[B7] Bonaz B. Sinniger V. Pellissier S. (2017). The vagus nerve in the neuro-immune Axis: Implications in the pathology of the gastrointestinal tract. Front. Immunol. 8, 1452. 10.3389/fimmu.2017.01452 29163522 PMC5673632

[B8] Brognara L. Cauli O. (2020). Mechanical plantar foot stimulation in Parkinson's disease: A scoping review. Diseases 8 (2). 10.3390/diseases8020012 PMC7349899 32397588

[B9] Colotta F. Dower S. K. Sims J. E. Mantovani A. (1994). The type II 'decoy' receptor: A novel regulatory pathway for interleukin 1. Immunol. Today 15 (12), 562–566. 10.1016/0167-5699(94)90217-8 7848516

[B10] Docagne F. Campbell S. J. Bristow A. F. Poole S. Vigues S. Guaza C. (2005). Differential regulation of type I and type II interleukin-1 receptors in focal brain inflammation. Eur. J. Neurosci. 21 (5), 1205–1214. 10.1111/j.1460-9568.2005.03965.x 15813930

[B11] Dujmovic I. Mangano K. Pekmezovic T. Quattrocchi C. Mesaros S. Stojsavljevic N. (2009). The analysis of IL-1 beta and its naturally occurring inhibitors in multiple sclerosis: The elevation of IL-1 receptor antagonist and IL-1 receptor type II after steroid therapy. J. Neuroimmunol. 207 (1-2), 101–106. 10.1016/j.jneuroim.2008.11.004 19162335

[B12] Ericsson A. Arias C. Sawchenko P. E. (1997). Evidence for an intramedullary prostaglandin-dependent mechanism in the activation of stress-related neuroendocrine circuitry by intravenous interleukin-1. J. Neurosci. 17 (18), 7166–7179. 10.1523/jneurosci.17-18-07166.1997 9278551 PMC6573255

[B13] Furlan R. Ardizzone S. Palazzolo L. Rimoldi A. Perego F. Barbic F. (2006). Sympathetic overactivity in active ulcerative colitis: Effects of clonidine. Am. J. Physiol. Regul. Integr. Comp. Physiol. 290 (1), R224–R232. 10.1152/ajpregu.00442.2005 16123227

[B14] Furlan R. Porta A. Costa F. Tank J. Baker L. Schiavi R. (2000). Oscillatory patterns in sympathetic neural discharge and cardiovascular variables during orthostatic stimulus. Circulation 101 (8), 886–892. 10.1161/01.cir.101.8.886 10694528

[B15] Garlind A. Brauner A. Höjeberg B. Basun H. Schultzberg M. (1999). Soluble interleukin-1 receptor type II levels are elevated in cerebrospinal fluid in Alzheimer's disease patients. Brain Res. 826 (1), 112–116. 10.1016/s0006-8993(99)01092-6 10216202

[B16] Hasty F. García G. Dávila C. H. Wittels S. H. Hendricks S. Chong S. (2020). Heart rate variability as a possible predictive marker for acute inflammatory response in COVID-19 patients. Mil. Med. 186 (1-2), e34–e38. 10.1093/milmed/usaa405 PMC7717314 33206183

[B17] Heart rate variability: Standards of measurement, physiological interpretation and clinical use. Task force of the European society of cardiology and the north American society of pacing and electrophysiology. Circulation. 1996;93(5):1043–1065. 8598068

[B18] Heimrich K. G. Lehmann T. Schlattmann P. Prell T. (2021). Heart rate variability analyses in Parkinson's disease: A systematic review and meta-analysis. Brain Sci. 11 (8). 10.3390/brainsci11080959 PMC8394422 34439578

[B19] Hoehn M. M. Yahr M. D. (1967). Parkinsonism: Onset, progression and mortality. Neurology 17 (5), 427–442. 10.1212/wnl.17.5.427 6067254

[B20] Huang Y. Smith D. E. Ibáñez-Sandoval O. Sims J. E. Friedman W. J. (2011). Neuron-specific effects of interleukin-1β are mediated by a novel isoform of the IL-1 receptor accessory protein. J. Neurosci. 31 (49), 18048–18059. 10.1523/jneurosci.4067-11.2011 22159118 PMC3261076

[B21] Hughes A. J. Daniel S. E. Kilford L. Lees A. J. (1992). Accuracy of clinical diagnosis of idiopathic Parkinson's disease: A clinico-pathological study of 100 cases. J. Neurol. Neurosurg. Psychiatry 55 (3), 181–184. 10.1136/jnnp.55.3.181 1564476 PMC1014720

[B22] Jouvenne P. Vannier E. Dinarello C. A. Miossec P. (1998). Elevated levels of soluble interleukin-1 receptor type II and interleukin-1 receptor antagonist in patients with chronic arthritis: Correlations with markers of inflammation and joint destruction. Arthritis Rheum. 41 (6), 1083–1089. 10.1002/1529-0131(199806)41:6<1083:aid-art15>3.0.co;2-9 9627018

[B23] Kenney M. J. Ganta C. K. (2014). Autonomic nervous system and immune system interactions. Compr. Physiol. 4 (3), 1177–1200. 10.1002/cphy.c130051 24944034 PMC4374437

[B24] Khazim K. Azulay E. E. Kristal B. Cohen I. (2018). Interleukin 1 gene polymorphism and susceptibility to disease. Immunol. Rev. 281 (1), 40–56. 10.1111/imr.12620 29247999

[B25] Li Y. Wang J. Li X. Jing W. Omorodion I. Liu L. (2021). Association between heart rate variability and Parkinson's disease: A meta-analysis. Curr. Pharm. Des. 27 (17), 2056–2067. 10.2174/1871527319666200905122222 32888281

[B26] Lindqvist D. Kaufman E. Brundin L. Hall S. Surova Y. Hansson O. (2012). Non-motor symptoms in patients with Parkinson's disease - correlations with inflammatory cytokines in serum. PLoS One 7 (10), e47387. 10.1371/journal.pone.0047387 23082161 PMC3474801

[B27] Liu X. Quan N. (2018). Microglia and CNS interleukin-1: Beyond immunological concepts. Front. Neurol. 9, 8. 10.3389/fneur.2018.00008 29410649 PMC5787061

[B28] Mantovani A. Dinarello C. A. Molgora M. Garlanda C. (2019). Interleukin-1 and related cytokines in the regulation of inflammation and immunity. Immunity 50 (4), 778–795. 10.1016/j.immuni.2019.03.012 30995499 PMC7174020

[B29] Mantovani A. Muzio M. Ghezzi P. Colotta F. Introna M. (1996). Negative regulators of the interleukin-1 system: Receptor antagonists and a decoy receptor. Int. J. Clin. Lab. Res. 26 (1), 7–14. 10.1007/bf02644768 8739850

[B30] Martelli D. Yao S. T. McKinley M. J. McAllen R. M. (2014). Reflex control of inflammation by sympathetic nerves, not the vagus. J. Physiol. 592 (7), 1677–1686. 10.1113/jphysiol.2013.268573 24421357 PMC3979618

[B31] Menza M. Dobkin R. D. Marin H. Mark M. H. Gara M. Bienfait K. (2010). The role of inflammatory cytokines in cognition and other non-motor symptoms of Parkinson's disease. Psychosomatics 51 (6), 474–479. 10.1176/appi.psy.51.6.474 21051678 PMC2987579

[B32] Molgora M. Supino D. Mantovani A. Garlanda C. (2018). Tuning inflammation and immunity by the negative regulators IL-1R2 and IL-1R8. Immunol. Rev. 281 (1), 233–247. 10.1111/imr.12609 29247989 PMC5922415

[B33] Müller B. Peri G. Doni A. Perruchoud A. P. Landmann R. Pasqualini F. (2002). High circulating levels of the IL-1 type II decoy receptor in critically ill patients with sepsis: Association of high decoy receptor levels with glucocorticoid administration. J. Leukoc. Biol. 72 (4), 643–649. 10.1189/jlb.72.4.643 12377932

[B34] Nance D. M. Sanders V. M. (2007). Autonomic innervation and regulation of the immune system (1987-2007). Brain Behav. Immun. 21 (6), 736–745. 10.1016/j.bbi.2007.03.008 17467231 PMC1986730

[B35] Pagani M. Lombardi F. Guzzetti S. Rimoldi O. Furlan R. Pizzinelli P. (1986). Power spectral analysis of heart rate and arterial pressure variabilities as a marker of sympatho-vagal interaction in man and conscious dog. Circ. Res. 59 (2), 178–193. 10.1161/01.res.59.2.178 2874900

[B36] Pavlov V. A. Tracey K. J. (2004). Neural regulators of innate immune responses and inflammation. Cell Mol. Life Sci. 61 (18), 2322–2331. 10.1007/s00018-004-4102-3 15378203 PMC11138906

[B37] Pavlov V. A. Tracey K. J. (2005). The cholinergic anti-inflammatory pathway. Brain Behav. Immun. 19 (6), 493–499. 10.1016/j.bbi.2005.03.015 15922555

[B38] Pavlov V. A. Wang H. Czura C. J. Friedman S. G. Tracey K. J. (2003). The cholinergic anti-inflammatory pathway: A missing link in neuroimmunomodulation. Mol. Med. 9 (5-8), 125–134. 10.1007/bf03402177 14571320 PMC1430829

[B39] Poewe W. (2008). Non-motor symptoms in Parkinson's disease. Eur. J. Neurol. 15 (1), 14–20. 10.1111/j.1468-1331.2008.02056.x 18353132

[B40] Pomeranz B. Macaulay R. J. Caudill M. A. Kutz I. Adam D. Gordon D. (1985). Assessment of autonomic function in humans by heart rate spectral analysis. Am. J. Physiol. 248 (1), H151–H153. 10.1152/ajpheart.1985.248.1.H151 3970172

[B41] Pongratz G. Straub R. H. (2014). The sympathetic nervous response in inflammation. Arthritis Res. Ther. 16 (6), 504. 10.1186/s13075-014-0504-2 25789375 PMC4396833

[B42] Qin X. Y. Zhang S. P. Cao C. Loh Y. P. Cheng Y. (2016). Aberrations in peripheral inflammatory cytokine levels in Parkinson disease: A systematic review and meta-analysis. JAMA Neurol. 73 (11), 1316–1324. 10.1001/jamaneurol.2016.2742 27668667

[B43] Reale M. Iarlori C. Thomas A. Gambi D. Perfetti B. Di Nicola M. (2009). Peripheral cytokines profile in Parkinson's disease. Brain Behav. Immun. 23(1), 55–63. 10.1016/j.bbi.2008.07.003 18678243

[B44] Reyes del Paso G. A. Langewitz W. Mulder L. J. van Roon A. Duschek S. (2013). The utility of low frequency heart rate variability as an index of sympathetic cardiac tone: A review with emphasis on a reanalysis of previous studies. Psychophysiology 50 (5), 477–487. 10.1111/psyp.12027 23445494

[B45] Rocha N. P. de Miranda A. S. Teixeira A. L. (2015). Insights into neuroinflammation in Parkinson's disease: From biomarkers to anti-inflammatory based therapies. Biomed. Res. Int. 2015, 628192. 10.1155/2015/628192 26295044 PMC4532803

[B46] Sharabi Y. Vatine G. D. Ashkenazi A. (2021). Parkinson's disease outside the brain: Targeting the autonomic nervous system. Lancet Neurol. 20 (10), 868–876. 10.1016/s1474-4422(21)00219-2 34536407

[B47] Singh P. Hawkley L. C. McDade T. W. Cacioppo J. T. Masi C. M. (2009). Autonomic tone and C-reactive protein: A prospective population-based study. Clin. Auton. Res. 19 (6), 367–374. 10.1007/s10286-009-0019-0 19504232 PMC2783459

[B48] Supino D. Minute L. Mariancini A. Riva F. Magrini E. Garlanda C. (2022). Negative regulation of the IL-1 system by IL-1R2 and IL-1R8: Relevance in pathophysiology and disease. Front. Immunol. 13, 804641. 10.3389/fimmu.2022.804641 35211118 PMC8861086

[B49] Tansey M. G. Wallings R. L. Houser M. C. Herrick M. K. Keating C. E. Joers V. (2022). Inflammation and immune dysfunction in Parkinson disease. Nat. Rev. Immunol. 2022. 10.1038/s41577-022-00684-6 PMC8895080 35246670

[B50] Tracey K. J. (2007). Physiology and immunology of the cholinergic antiinflammatory pathway. J. Clin. Invest. 117 (2), 289–296. 10.1172/jci30555 17273548 PMC1783813

[B51] Tracey K. J. (2002). The inflammatory reflex. Nature 420 (6917), 853–859. 10.1038/nature01321 12490958

[B52] Williams D. P. Koenig J. Carnevali L. Sgoifo A. Jarczok M. N. Sternberg E. M. (2019). Heart rate variability and inflammation: A meta-analysis of human studies. Brain Behav. Immun. 80, 219–226. 10.1016/j.bbi.2019.03.009 30872091

[B53] Yao S. Chan A. Elsaafien K. Korim W. (2020). The inflammatory cytokine, interleukin‐1‐beta, drives central immune cell infiltration, changes in sympathetic nerve activity and blood pressure. FASEB J. 34, 1. 10.1096/fasebj.2020.34.s1.04682

[B54] Zamunér A. R. Shiffer D. Barbic F. Minonzio M. Andrade C. P. Corato M. (2019). Mechanical somatosensory stimulation decreases blood pressure in patients with Parkinson's disease. J. Hypertens. 37 (8), 1714–1721. 10.1097/HJH.0000000000002084 31107357

